# The Relationship Between Parenting Styles and Children’s Prosocial Behavior: The Mediating Role of Children’s Emotional Intelligence

**DOI:** 10.3390/bs16010155

**Published:** 2026-01-22

**Authors:** Siqi Zhang, Ping Wang, Weichen Wang, Heng Su, Xianbing Zhang

**Affiliations:** 1 Faculty of Education, Northeast Normal University, Changchun 130024, China; zhangsq541@nenu.edu.cn (S.Z.); wangp@nenu.edu.cn (P.W.); suheng705@nenu.edu.cn (H.S.); 2 School of Psychology, Northeast Normal University, Changchun 130024, China; wangwc036@nenu.edu.cn

**Keywords:** parenting styles, prosocial behavior, emotional intelligence

## Abstract

Prosocial behavior is an important manifestation of socialization in young children. As the primary setting for socialization of young children, the family bears the significant responsibility of fostering prosocial behavior in young children. Drawing on family systems theory and Goleman’s emotional intelligence theory, the purpose of this study was to investigate the relationship between parenting styles and children’s prosocial behavior and the mediating role of children’s emotional intelligence in it. In this study, an online questionnaire was distributed to 869 young children’s parents using the Parenting Style Questionnaire, Children’s Prosocial Behavior Questionnaire, and Children’s Emotional Intelligence Questionnaire. The results indicated that democratic parenting style positively influenced children’s prosocial behavior, while indulgent parenting style, permissive parenting style and inconsistent parenting style negatively impacted it. Authoritarian parenting style had no significant effect on children’s prosocial behavior. Children’s emotional intelligence mediated the relationship between parenting styles and prosocial behavior. This study explored factors influencing children’s prosocial behavior from both external family systems and internal individual perspectives and revealed their underlying mechanisms, providing theoretical support for research and educational practice on children’s prosocial behavior.

## 1. Introduction

Prosocial behavior is a crucial indicator of an individual’s socialization and plays a vital role in their social development (Nie et al., 2025). Early childhood prosocial behavior refers to children’s spontaneous expressions of concern and empathy toward others during social interactions, manifesting in actions beneficial to others and society, such as cooperation, sharing, helping, and caring for others (J. P. Li et al., 2024). As a positive form of social behavior, children’s prosocial behavior which is a vital component of social domain education, promotes the development of their prosocial norms and habits. Early development of prosocial behavior profoundly influences subsequent mental health (Liu et al., 2022), peer relationships, interpersonal interactions (Meulenbeek et al., 2025), and future development (Y. J. Wang et al., 2020).

Both instability and plasticity characterize the development of prosocial behavior in early childhood (Xie et al., 2024), making this stage the optimal period for its cultivation. The establishment of prosocial behavior patterns during this phase determines the trajectory of altruistic behavior in adulthood while laying the foundation for self-awareness, interpersonal relationships, and social learning (W. Zhang et al., 2022). Evidently, focusing on and nurturing children’s prosocial behavior during early childhood is a practical necessity for their socialization. The development of prosocial behavior significantly impacts children’s physical and mental health, as well as the expression of their knowledge and abilities, making it a cornerstone of their social development. Throughout a child’s growth, the development of prosocial behavior is influenced by both external factors and individual internal factors. Simultaneously, as cognitive abilities advance, prosocial behavior increases rapidly during early childhood (Yavuz et al., 2022). However, by middle to late childhood, behavioral trajectories diverge: prosocial behavior may either gradually increase (H. Li et al., 2025) or decrease (Katsantonis & McLellan, 2024), with heterogeneous development observed across different behavioral subtypes. Therefore, to enhance the level of prosocial behavior development and promote children’s social interaction skills, it is both necessary and urgent to investigate the influencing factors and underlying mechanisms of prosocial behavior in young children.

### 1.1. Parenting Styles and Children’s Prosocial Behavior

External environments influence the development of individual prosocial behavior. The family is the earliest setting for a child’s socialization process and a significant external factor influencing the development of prosocial behavior (Liang et al., 2025). According to ecological systems theory, environmental background plays a crucial role in individual development. This theory posits that individual development is embedded within a four-layered networked environmental system, with the family being the innermost microsystem and the direct environment for individual activities and interactions, which has a significant impact on individual development (Hai, 2024).

Parenting styles represent relatively stable behavioral tendencies exhibited by parents during child-rearing, reflecting their educational philosophies and habitual parenting practices (Cheng et al., 2025). Parents, as central components of the family, directly or indirectly influence children’s social development through their parenting styles and educational philosophies, thereby shaping the development of children’s prosocial behavior (J. Z. Zhang et al., 2022). Family systems theory posits that subsystems within the family are interconnected and interacted with one another, and parental parenting constitutes a vital component of the parent–child subsystem. Parental behavior during child-rearing can alter subsequent child development and influence changes in the parent–child subsystem and even the entire family system structure (Y. J. Peng et al., 2023). Simultaneously, as an emotional unit, the family’s interpersonal emotional bonds and interaction patterns profoundly shape early childhood development. The Emotional Spillover Hypothesis suggests that parents’ negative emotions and behaviors may “spill over” into parent–child relationships, which can have a negative impact on the development of young children through negative parent–child interactions, hindering their emotional intelligence and prosocial behavior (Calatrava et al., 2022). In this study, parenting styles are defined as significant external environmental factors influencing children’s prosocial behavior, and in light of this, Hypothesis 1 was proposed: Democratic parenting style exerts a positive influence on children’s prosocial behavior, while indulgent, permissive, authoritarian and inconsistent parenting styles exert negative influences.

### 1.2. The Mediating Role of Children’s Emotional Intelligence

Early childhood emotional intelligence refers to a child’s ability to process emotional information and manage emotional issues (Pumyoch & Srikoon, 2024). Goleman’s theory of emotional intelligence posits that children’s emotional intelligence serves as a crucial foundation for prosocial behavior development, with parenting styles being a key factor influencing this growth (Goleman, 1995/2010). The critical window for emotional intelligence development occurs in the first few years after birth. Research indicates that key emotional experiences during the first four years of life exert profound and enduring effects (Kang & Guo, 2021). Consequently, if parents consistently interact with infants using a specific pattern, fundamental emotional experiences become ingrained in the child (S. T. Ma et al., 2021). Moreover, differences in emotional development are linked to the family environment, with parenting styles playing a significant role (X. Yang & Song, 2025). Positive parenting styles provide children with warm emotional support, encouraging proactive communication with parents and enhancing emotional intelligence (Kang & Guo, 2021). Conversely, harsh parenting styles correlate with emotional and behavioral issues in children, leading to withdrawn and avoidant behaviors that hinder the development of prosocial conduct (Ping et al., 2023). Therefore, different parenting styles can instill distinct emotional perceptions in children, influencing their perceptions of themselves and their closest relationships, thereby shaping emotional intelligence development. Based on these analyses, Hypothesis 2 was proposed: Democratic parenting style exerts a positive influence on children’s emotional intelligence, while indulgent, permissive, authoritarian and inconsistent parenting styles exert negative influences.

Emotional cognition is highly social, prompting increased attention to the variability in emotional development and its impact on children’s social development (Su & Ding, 2025). It has been found that children’s emotional intelligence exhibits a high predictive effect on prosocial behavior performance, with children demonstrating stronger emotional intelligence development exhibiting more prosocial behavior (Wu et al., 2023). As a crucial component of children’s social and emotional development, children with high emotional intelligence can accurately understand and respond to their own and others’ emotions, demonstrate emotional wisdom in social interactions, and enhance relationships with others (Q. F. Peng et al., 2025). Goleman’s theory of emotional intelligence posits that the development of children’s emotional intelligence encompasses skills such as managing interpersonal relationships. In the process of developing emotional intelligence, children’s empathy, communication abilities, and cooperative skills all advance, laying the groundwork for the emergence and development of prosocial behavior. Only when children possess strong emotional intelligence can they genuinely recognize and respond to others’ emotions, actively helping peers regulate and manage negative feelings. Such children are also better at emotional resonance, more willing to communicate and cooperate with peers, and thus exhibit greater prosocial behavior (Goleman, 1995/2010).

External factors often influence individuals through the mediation of certain internal factors. Children’s emotional intelligence constitutes an internal factor and a subjective determinant influencing the development of their prosocial behavior. Parenting styles, as external factors, influence the development of prosocial behavior through the internal factor of emotional intelligence, consistent with previous research findings (Akgöz et al., 2025). Goleman’s theory of emotional intelligence suggests that emotional potential acts as a mediating capacity in the development of prosocial behavior. Positive parenting styles enhance children’s well-being by improving their ability to express and regulate emotions, fostering secure attachments with parents, and thereby promoting prosocial behavior (Goleman, 1995/2010). Based on this analysis, this study posits a significant correlation among parenting styles, children’s prosocial behavior, and emotional intelligence, and in light of this, Hypothesis 3 was proposed: Children’s emotional intelligence exerts a significant positive influence on their prosocial behavior and mediates the relationship between parenting styles and children’s prosocial behavior.

### 1.3. Current Research

**Figure 1:**
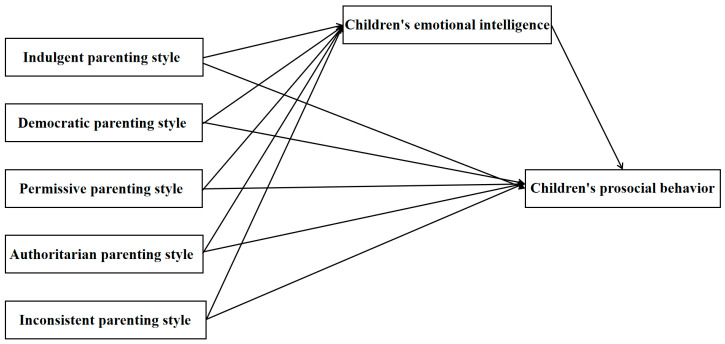
Hypothesis model.

This study, grounded in family systems theory and Goleman’s emotional intelligence theory, employs a questionnaire survey to examine the relationship between parenting styles and children’s prosocial behavior, as well as the mediating role of children’s emotional intelligence in this relationship. The research aims to reveal the factors influencing children’s prosocial behavior and their underlying mechanisms, providing important insights for enhancing children’s prosocial behavior and promoting their comprehensive development. The study proposes the following hypotheses (Figure 1):

**Hypothesis** **1.** *Democratic parenting style exerts a positive influence on children’s prosocial behavior, while indulgent, permissive, authoritarian, and inconsistent parenting styles exert negative influences*.

**Hypothesis** **2.** *Democratic parenting style exerts a positive influence on children’s emotional intelligence, while indulgent, permissive, authoritarian and inconsistent parenting styles exert negative influences*.

**Hypothesis** **3.** *Children’s emotional intelligence exerts a significant positive influence on their prosocial behavior and mediates the relationship between parenting styles and children’s prosocial behavior*.

## 2. Materials and Methods

### 2.1. Participants

An online questionnaire was distributed to parents of children aged 3–5 years across different provinces of China. One primary caregiver (father or mother) was invited to complete the survey. At the same time, this study selected physically and mentally healthy children from public kindergarten as the research subjects, taking into account their physical and mental development when selecting participants to minimize differences between samples. Based on an expected effect size of 0.20 and a required statistical power of 0.80, a prior power analysis indicated that a structural equation model involving 3 latent variables and 16 observed variables would require at least 296 participants (Soper, 2025; https://www.danielsoper.com/statcalc, accessed on 15 March 2025). A total of 1005 questionnaires were distributed. The questionnaire used the Wenjuanxing online receipt (Changsha Ranxing Information Technology Co., Ltd., Changsha, Hunan, China; https://www.wjx.cn/), and all questions filled were mandatory, ensuring no missing data. After excluding 136 invalid responses (due to carelessness, etc.), 869 valid questionnaires were obtained, yielding a response rate of 86.47%. Demographic characteristics of the study participants are presented in Table 1.

### 2.2. Measures

#### 2.2.1. Demographic Questionnaire

Based on prior research, demographic factors influencing parenting styles, children’s prosocial behavior, and emotional intelligence were identified. Considering the overall questionnaire structure, the most impactful demographic factors were selected to form the basic information section. This includes child gender, child age, whether the child is an only child, parents’ educational background, and the average monthly salary of parents. The survey includes both basic information about the children, such as their age, and basic information about the questionnaire respondents, such as their educational background.

#### 2.2.2. Parenting Style Questionnaire

Based on existing research by Schaefer (1959) and Maccoby (1988), L. Z. Yang and Yang (1998) developed the Parenting Style Questionnaire adapted to the Chinese sociocultural context. The scale consists of 40 items and includes five dimensions: (1) Indulgent parenting style, which refers that parents indulge children’s every request and cater to their every whim (7 items, e.g., “The child gets whatever he/she wants”); (2) Democratic parenting style, which refers that parents respect their child and interact on equal terms (10 items, e.g., “Develop the child’s talents based on their own interests”); (3) Permissive parenting style, which refers that parents adopt a laissez-faire attitude toward their child’s upbringing (9 items, e.g., “Do not concern myself with the child’s daily affairs”); (4) Authoritarian parenting style, which refers that parents intervene and control their children (8 items, e.g., “Requiring children to report everything they do to parents”); (5) Inconsistent parenting style, which refers that parents lack consistency in their child-rearing approach (6 items, e.g., “Allowing or refusing the same thing at different times”). A 5-point Likert scale was used (1 = Never, 2 = Rarely, 3 = Sometimes, 4 = Often, 5 = Always), with a higher score indicating a more prominent parenting style. This multilevel scoring system captures the distribution of scores across different parenting styles, overcoming the limitations of single-dimensional categorization. Consequently, all five parenting style dimensions were incorporated into the subsequent analysis model. The overall Cronbach’s α coefficient for this scale in this study was 0.873, with subscale Cronbach’s α coefficients of 0.830, 0.811, 0.823, 0.851, and 0.835.

#### 2.2.3. Prosocial Behavior Questionnaire

The Prosocial Behavior Questionnaire developed by Deng (2013) was employed. The scale consists of 28 items and includes five dimensions: (1) Cooperation, which refers that children joint task participation with others (9 items, e.g., “Completing crafts together with classmates”); (2) Sharing, which refers that children share possessions with peers (7 items, e.g., “Willingly sharing food with parents or nearby individuals”); (3) Comforting, which refers that children console others when they are upset or hurt (4 items, e.g., “The child actively comforts other children who are sad or crying while playing together”); (4) Helping, which refers that children assist others or the group (4 items, e.g., “The child often volunteers to help others”); (5) Public Virtue, which refers that children actively make beneficial contributions for collective interests (4 items, e.g., “Treat public property with care”). A 5-point Likert scale was used (1 = Strongly Disagree, 2 = Somewhat Disagree, 3 = Undecided, 4 = Somewhat Agree, 5 = Strongly Agree), with a higher score indicating a more prominent prosocial behavior. In this study, the Cronbach’s α coefficient for this scale was 0.933, with subscale Cronbach’s α coefficients of 0.802, 0.806, 0.837, 0.804, and 0.792.

#### 2.2.4. Emotional Intelligence Questionnaire

The Emotional Intelligence Questionnaire developed by R. R. Li (2012) was employed. The scale consists of 25 items and includes six dimensions: (1) Perceiving and Experiencing One’s Own Emotions, which refers to the ability to recognize and understand one’s own emotional responses (3 items, e.g., “When taking the child on an outing, he/she says they are having a lot of fun”); (2) Perceiving and Experiencing Others’ Emotions, which refers to the ability to recognize and understand others’ emotional states (6 items, e.g., “When family members return home smiling, he can tell they are happy by their expressions”); (3) Expressing and Evaluating One’s Own Emotions, which refers to the ability to express and assess one’s own emotional responses (3 items, e.g., “When wronged, he can clearly express his dissatisfaction to others”); (4) Expressing and Evaluating Others’ Emotions, which refers to the ability to express and assess others’ emotional states (3 items, e.g., “When seeing another child fall and bleed, he feels sorry for them and says ‘Poor thing!’”); (5) Regulating and Controlling One’s Own Emotions, which refers to the ability to regulate and control one’s own emotional responses (5 items, e.g., “When the child makes a mistake and is criticized, he can accept the criticism and actively correct it”); (6) Regulating and Controlling Others’ Emotions, which refers to the ability to regulate and control others’ emotional responses (5 items, e.g., “When family members argue, the child will try to mediate their emotions”). A 6-point Likert scale was used (1 = Never, 2 = Rarely, 3 = Occasionally, 4 = Sometimes, 5 = Often, 6 = Always), with a higher score indicating a more prominent emotional intelligence. In this study, the Cronbach’s α coefficient for this scale was 0.927, with subscale Cronbach’s α coefficients of 0.788, 0.864, 0.806, 0.812, 0.803, and 0.833.

### 2.3. Data Collection and Processing Methods

Parents of children aged 3–5 years were recruited via an online platform, and they completed self-report assessments of their parenting behaviors and filled out the Children’s Prosocial Behavior Questionnaire and Children’s Emotional Intelligence Questionnaire on behalf of their children. After screening for identical responses and abnormally long or short completion times, 869 valid questionnaires were retained. Subsequently, analysis of demographic differences was conducted using SPSS 25.0 (IBM Corporation, Armonk, NY, USA) for all variables to investigate whether children’s prosocial behavior and its sub-dimensions differed across demographic variables. Specifically, a *t*-test was used to examine the child gender and whether the child is an only child, while one-way ANOVA was used to analyze the child age, parents’ education background, and average monthly salary of parents. Results with significant differences were included in subsequent analyses. Finally, structural equation modeling (SEM) was performed with Amos 27.0 (IBM Corporation, Armonk, NY, USA) to test the mediating effects of emotional intelligence across five parenting style models. Each model used one of the parenting styles (indulgent, democratic, permissive, authoritarian, or inconsistent) as the independent variable, children’s prosocial behavior as the dependent variable, and children’s emotional intelligence as the mediating variable. SEM was applied to test the mediating effects.

## 3. Results

### 3.1. Common Method Bias and Multicollinearity Tests

All data in this study were derived from participants’ self-reports, potentially introducing systematic variance bias. Exploratory factor analysis with Harman’s single-factor test was conducted to assess latent common method bias. Results extracted 16 principal components with eigenvalues exceeding 1. The first principal component explained 19.72% of the variance, below the 40% critical threshold, indicating no significant common method bias (H. Zhou & Long, 2004). Additionally, to assess potential multicollinearity among predictor variables, a variance inflation factor (VIF) analysis was conducted. All independent variables exhibited VIF values below 5, confirming the absence of multicollinearity (Jia et al., 2018).

### 3.2. Demographic Differences in Children’s Prosocial Behavior

This study employed independent samples *t*-test and one-way ANOVA to analyze differences in children’s prosocial behavior across demographic variables. At the same time, the effect sizes of demographic variables were calculated using the psychometrica website to determine their practical significance for the dependent variable (Lenhard & Lenhard, 2022; https://www.psychometrica.de/effect_size.html, accessed on 22 May 2025). The results showed no significant difference in prosocial behavior by only-child status (*t* = −1.018, *p* = 0.309, Cohen’s *d* = 0.07, 95%CI [−0.064, 0.204]) or parental average monthly income (*F* = 0.652, *p* = 0.625, *η*^2^ = 0.003, 95%CI [0.000, 0.009]). Significant differences were found in prosocial behavior based on child gender (*t* = −2.161, *p* = 0.031, Cohen’s *d* = 0.138, 95%CI [0.004, 0.272]), child age (*F* = 7.750, *p* < 0.001, *η*^2^ = 0.018, 95%CI [0.005, 0.035]), and parents’ educational background (*F* = 4.640, *p* < 0.01, *η*^2^ = 0.011, 95%CI [0.001, 0.026]), as detailed in Table 2 and Table 3. Specifically, girls exhibited significantly higher prosocial behavior tendencies than boys. This tendency was reflected in various dimensions of children’s prosocial behavior. Simple effects analysis revealed that the prosocial behavior of 4-year-old and 5-year-old children was significantly higher than that of 3-year-old children (*p* < 0.001). Specifically, 4-year-old and 5-year-old children exhibited more sharing behavior than 3-year-old children (*p* < 0.001), and there were significant differences in the dimensions of comforting (*p* < 0.001) and public virtue (*p* < 0.01) among children of different ages. Furthermore, children whose parents had college degree showed significantly higher prosocial behavior than those whose parents had bachelor’s degree and above (*p* = 0.003) or high school or vocational diploma (*p* = 0.038). Specifically, children whose parents had college degree exhibited more cooperative, helpful, and ethical behaviors (*p* < 0.01). Children whose parents had bachelor’s degree and above exhibited more helpful behavior than those whose parents had high school or vocational diploma (*p* = 0.011).

### 3.3. Descriptive Statistics and Correlation Analysis of Variables

Descriptive statistics and correlation analysis results for each variable are presented in Table 4. Findings indicated significant pairwise correlations among parenting styles, children’s prosocial behavior, and children’s emotional intelligence. Democratic parenting style, children’s prosocial behavior, and children’s emotional intelligence showed significant positive correlations (*r* = 0.331, *r* = 0.332, *ps* < 0.01). Indulgent, permissive, authoritarian, and inconsistent parenting styles showed significant negative correlations with children’s prosocial behavior (*r* = −0.219, *r* = −0.267, *r* = −0.160, *r* = −0.231, *ps* < 0.01) and emotional intelligence (*r* = −0.165, *r* = −0.204, *r* = −0.145, *r* = −0.181, *ps* < 0.01). Additionally, children’s emotional intelligence showed a significant positive correlation with their prosocial behavior (*r* = 0.605, *p* < 0.01).

### 3.4. Mediating Effect of Children’s Emotional Intelligence

**Figure 2:**
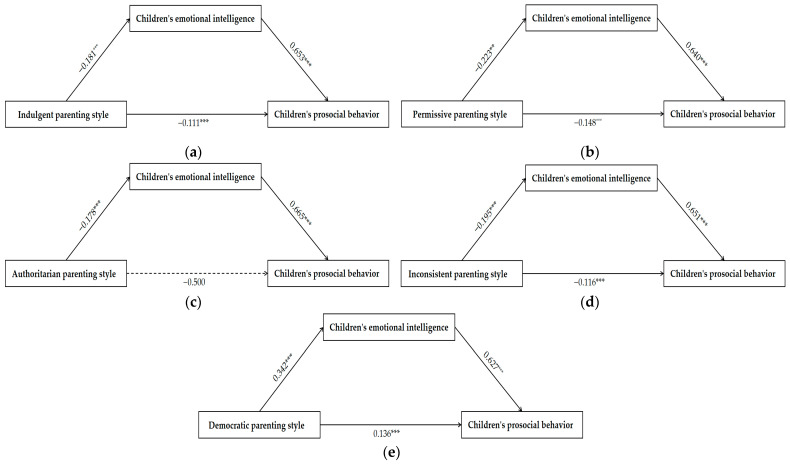
Mediation effect diagram. (**a**) The mediating effect of indulgent parenting style; (**b**) the mediating effect of permissive parenting style; (**c**) the mediating effect of authoritarian parenting style; (**d**) the mediating effect of inconsistent parenting style; (**e**) the mediating effect of democratic parenting style (** *p* < 0.01, *** *p* < 0.001).

In order to further explore the mechanisms underlying the influence of parenting styles on prosocial behavior, in this study, the mediating role of emotional intelligence in this relationship was investigated. In this study, the five parenting styles were treated as unidimensional constructs, with their respective means directly entered into the model. Children’s emotional intelligence and prosocial behavior encompassed multiple dimensions, so the mean of each dimension was included in the model. Based on demographic variable analysis results, child gender, child age, and parents’ educational background were incorporated as control variables. The result showed that the five models had acceptable fit indices (Table 5). The specific results are shown in Figure 2 and Table 6. SEM results showed that democratic parenting style exerted a positive influence on prosocial behavior (*β* = 0.136, *p* < 0.001). Indulgent parenting style (*β* = −0.111, *p* < 0.001), permissive parenting style (*β* = −0.148, *p* < 0.001), and inconsistent parenting style (*β* = −0.116, *p* < 0.001) exerted a negative influence on children’s prosocial behavior. Authoritarian parenting style had no significant effect on children’s prosocial behavior (*β* = −0.500), while children’s emotional intelligence exerted a significant positive influence on prosocial behavior (*β* = 0.627, *β* = 0.653, *β* = 0.640, *β* = 0.665, *β* = 0.651, *ps* < 0.001).

## 4. Discussion

This study, grounded in family systems theory and Goleman’s emotional intelligence theory, examined the relationships among parenting styles, children’s prosocial behavior, and children’s emotional intelligence. Results indicated that democratic parenting style positively influenced children’s prosocial behavior, while indulgent, permissive, and inconsistent parenting styles negatively affected it. The authoritarian parenting style showed no significant impact on prosocial behavior. Children’s emotional intelligence mediated the relationship between parenting styles and prosocial behavior. This study reveals key factors influencing children’s prosocial behavior, providing important insights for enhancing such behavior.

### 4.1. Interpretation of Findings

#### 4.1.1. Parenting Styles and Children’s Prosocial Behavior

The findings indicated that democratic parenting style positively influenced children’s prosocial behavior, while indulgent, permissive, and inconsistent parenting styles negatively impacted it. The results support Hypothesis 1, confirming that positive parenting styles promote the development of children’s prosocial behavior. The positive effect of democratic parenting style aligns with existing studies that state that parents’ words and actions during the child-rearing process subtly shape an individual’s behavioral habits, beliefs, and personality, thereby influencing their prosocial behavior (Pan et al., 2024; Zhao et al., 2022).

First, according to family systems theory, emotions and behaviors between parents and children are mutually influential. Positive parenting styles foster greater warmth, support, and cohesion within the family, enabling children to become more confident and autonomous, enhancing their ability to adapt to society and exhibit increased prosocial behavior (Zhao et al., 2022). Moreover, the family system exerts an immersive influence on the individual, characterized by strong intergenerational transmission, meaning that parents’ positive or negative parenting styles are transmitted to children through the parent–child relationship subsystem, allowing children to gradually acquire the emotional attitudes and behaviors demonstrated by their parents during child-rearing (Yu et al., 2023). Simultaneously, the spillover hypothesis posits that experiences acquired within one relationship extend to others (M. M. Li et al., 2019). Thus, not only do parents’ negative emotional attitudes and behaviors spill over into the parent–child subsystem, but negative experiences learned during upbringing also diffuse into children’s peer relationship subsystem, subsequently expanding to other peer relationships and thereby impacting the development of children’s prosocial behavior (J. Zhou et al., 2023). Second, Bandura’s social learning theory also highlights observation and imitation as crucial pathways for early childhood learning (Bandura, 1977/2015). Young children primarily develop behavioral patterns by observing and imitating those around them. Research indicates that parents or primary caregivers practicing democratic parenting style exhibit more exemplary behaviors, serving as models for children to emulate (L. F. Zhang et al., 2023). Last, within this warm and positive family environment, parents and children establish strong attachment bonds, fostering a sense of security in young children. This security promotes optimism, self-confidence, and the courage to explore their surroundings and interact with others, effectively handle conflicts with others, and present stronger interpersonal attraction among peers. They will exhibit more prosocial behavior (Lei et al., 2022).

In addition, the impact of authoritarian parenting style on prosocial behavior was not significant, which is consistent with the findings of W. J. Zhang and Zhu (2021). Within Chinese culture, emphasizing filial piety and collectivism, the “strict discipline” component of authoritarian parenting coexists with affection. Parents likely impose strict control out of love for their children, aligning with the characteristics of “affection-constrained” parenting. Consequently, the negative effects of authoritarian parenting style on early childhood development may be buffered by parental affection, complicating its relationship with children’s prosocial behavior.

#### 4.1.2. Mediating Role of Children’s Emotional Intelligence

The results of this study showed that parenting styles exerted a significant influence on children’s emotional intelligence, that children’s emotional intelligence exerted a significant positive influence on their prosocial behavior, and that children’s emotional intelligence partially mediated the relationship between parenting styles and children’s prosocial behavior. Specifically, the democratic parenting style exerted a positive influence on children’s emotional intelligence, which in turn promoted their prosocial behavior. This result validates Hypotheses 2 and 3.

First, in this study, it was found that democratic parenting style exerted a positive influence on children’s emotional intelligence, while indulgent, permissive, authoritarian and inconsistent parenting styles exerted negative influences, further validating previous research findings (Y. Y. Ma et al., 2023). Parenting styles are significantly correlated with children’s emotional intelligence. Positive parenting styles provide emotional warmth and understanding, reducing the frequency of abnormal emotions in children. Conversely, behaviors such as excessive punishment, indulgence, and distrust lead to more negative emotions in children, hindering the development of emotional intelligence. Democratic parenting style positively influences children’s emotional intelligence development. This approach fosters an equal and warm parenting environment, strengthens parent–child bonds, promotes secure attachment formation, and builds children’s self-confidence, thereby supporting healthy emotional development (Feldman et al., 2025). Additionally, parents employing the democratic parenting style are sensitive to children’s emotional shifts and respond positively to negative emotions. This supportive approach to negative feelings facilitates the development of positive emotion regulation strategies, thereby advancing emotional intelligence (Huang et al., 2024).

In contrast, in this study, it was also found that children’s emotional intelligence had a significant positive impact on their prosocial behavior, consistent with previous research findings (J. Wang & Wang, 2021). Many developmental psychologists have observed that early childhood experiences of emotional and social fulfillment influence subsequent behavioral development (Paz et al., 2022). When children possess higher emotional intelligence, they better recognize and express their own and others’ emotions, effectively resolving emotional issues for themselves or others (Feng & Wang, 2023). Conversely, when children frequently experience negative emotions like tension or anxiety, their acceptability among peers decreases, leading to more problem behaviors and hindering the development of prosocial behavior (Liu et al., 2020). Goleman’s emotional intelligence theory identifies emotional intelligence as a crucial factor in developing “people skills”-the social competence required for effective interpersonal relationships. These social skills help children improve relationships, motivate and inspire others, and persuade or comfort peers. Conversely, children lacking social skills struggle with interpersonal dynamics, face difficulties adapting to society, and experience hindered prosocial behavior development (Goleman, 1995/2010). Simultaneously, higher levels of emotional intelligence correlate with stronger interpersonal management abilities, enabling children to navigate diverse social contexts effectively. Such children exhibit heightened prosocial behavior in social interactions, fostering harmonious relationships (Casini et al., 2022).

In addition, this study indicated that children’s emotional intelligence mediated the relationship between parenting styles and prosocial behavior. According to Goleman’s theory of emotional intelligence, positive parenting styles foster children’s emotional awareness, understanding, and control abilities, thereby promoting the development of prosocial behavior (Goleman, 1995/2010). Positive parenting provides greater emotional and behavioral support, enhances children’s sense of security and interpersonal trust, fosters their happiness, and improves adaptive emotional regulation-ensuring positive emotional experiences and healthy psychological development. The level of children’s emotional intelligence determines their development in communication, cooperation, and interpersonal relationship management, directly influencing the level of their prosocial behavior.

### 4.2. Theoretical and Practical Implications

Prosocial behavior is an important manifestation of socialization in young children, and parenting styles exert a significant influence on the development of children’s prosocial behavior. This study aims to explore the relationship between parenting styles and children’s prosocial behavior. Results indicated that parenting styles not only have a direct effect on children’s prosocial behavior but also have an indirect effect on children’s prosocial behavior through children’s emotional intelligence. This study deepens our understanding of the underlying mechanisms linking parenting styles and children’s prosocial behavior, offering theoretical guidance for enhancing such behavior. On the one hand, the research helps to understand the current development status of children’s prosocial behavior and the underlying mechanisms of related influencing factors, especially the specific role of children’s emotional intelligence in it, allowing everyone to see the value of emotions in behavioral development and value the power of emotions. On the other hand, the research results demonstrate the crucial role of parents in the behavioral development of young children, highlighting the enormous value of family education. Consequently, it suggests that parents can improve their parenting styles, create a positive family atmosphere, and adopt appropriate methods to enhance children’s emotional intelligence development, strengthen their empathy and emotional regulation abilities, thereby promoting the development of children’s prosocial behavior.

### 4.3. Research Limitations and Future Directions

This study has certain limitations. First, the level of children’s prosocial behavior was reported by parents, potentially introducing subjective biases in the results. Future research may use experimental methods, such as the “out of reach” paradigm, moral dilemma experiments, and trust games, to measure children’s prosocial behavior. Experimental methods can better control variables and observe specific prosocial behavior in a short period of time, making the results more objective. Second, the measurement of children’s prosocial behavior in this study employed a cross-sectional design. Future research could adopt a longitudinal perspective to further reveal the causal relationships among variables and test the stability of the current relationships. At the same time, this study used a variable-centered approach to reveal the overall mediating mechanism between parenting styles, children’s emotional intelligence, and children’s prosocial behavior. However, we note that parenting styles may have a high degree of heterogeneity, and different families may combine different parenting models. Future research can use individual-centered latent profile analysis to classify parents’ parenting styles more finely and identify potential subgroups with different characteristics behind the data. On this basis, we further examine whether these different parenting profiles have differentiated impact patterns on children’s prosocial behavior through the mediating effect of children’s emotional intelligence. This method will help us provide more targeted guidance for family education from the perspective of “what types of parents” rather than just “a certain dimension of parental styles”. Furthermore, this study primarily focused on reports from one parent (usually the primary caregiver). In the future, information such as the parents’ gender and age could be included, and differential tests could be conducted to explore their impacts on various variables, thereby enriching the research content. Finally, this study validated independent mediation models of five dimensions of parenting styles influencing prosocial behavior through emotional intelligence, and explored the relationship between variables using children’s emotional intelligence as the sole mediator variable, which limited the exploratory nature of the model construction. Future research could construct a comprehensive model of five parenting styles and incorporate additional mediating variables, such as marital relationships or parent–child relationships, while also employing moderating variables to explore which factors might enhance the influence of parenting styles on children’s prosocial behavior. This would provide deeper insights into the relationship between parenting styles and children’s prosocial behavior.

## 5. Conclusions

This study examined the relationships among parenting styles, children’s prosocial behavior, and children’s emotional intelligence, as well as the mediating role of children’s emotional intelligence in this relationship. Democratic parenting style significantly promotes the development of children’s prosocial behavior, while indulgent, permissive, and inconsistent parenting styles hinder it. In this study, authoritarian parenting does not significantly influence children’s prosocial behavior. Furthermore, parenting styles not only have a direct effect on children’s prosocial behavior, but also have an indirect effect on children’s prosocial behavior through children’s emotional intelligence. This study reveals the mechanism through which parenting styles influence children’s prosocial behavior. Therefore, parents can foster prosocial behavior by adopting supportive parenting practices and enhancing children’s emotional intelligence.

## Figures and Tables

**Table 1:** Demographic Characteristics of Study Participants (*N* = 869).

Demographic Variable	Category	Number	Percentage (%)
Child Gender	Male	479	55.10
	Female	390	44.90
Child Age	3 years old	281	32.30
	4 years old	303	34.90
	5 years old	285	32.80
Whether the child is an only child	Yes	390	44.90
	No	479	55.10
Parents’ educational background	High school or vocational diploma	220	25.30
	College degree	247	28.40
	Bachelor’s degree and above	402	46.30
Average monthly salary of parents	5000 yuan and below	129	14.80
	5001–8000 yuan	246	28.30
	8001–12,000 yuan	210	24.20
	12,001–15,000 yuan	104	12.00
	Above 15,000 yuan	180	20.70

**Table 2:** Analysis of Differences in Children’s Prosocial Behavior on Child Gender, Child Age, and Parents’ Educational Background.

Demographic Variable	Category	*M ± SD*	*t* or *F*
Child Gender	Male	3.94 ± 0.47	−2.161 *
	Female	4.00 ± 0.47	
Child age	3 years old	3.88 ± 0.47	7.750 ***
	4 years old	4.00 ± 0.46	
	5 years old	4.02 ± 0.48	
Parents’ educational background	High school or vocational diploma	3.97 ± 0.47	4.640 **
	College degree	4.04 ± 0.47	
	Bachelor’s degree and above	3.96 ± 0.47	

Note. * *p* < 0.05, ** *p* < 0.01, *** *p* < 0.001.

**Table 3:** Analysis of Differences in Various Dimensions of Children’s Prosocial Behavior on Child Gender, Child Age, and Parents’ Educational Background.

	Demographic Variable	Category	*M ± SD*	*t* or *F*
Cooperation	Child Gender	Male	4.03 ± 0.50	−1.083
		Female	4.07 ± 0.52	
	Child age	3 years old	4.00 ± 0.51	2.289
		4 years old	4.07 ± 0.49	
		5 years old	4.08 ± 0.53	
	Parents’ educational background	High school or vocational diploma	3.99 ± 0.51	4.880 **
		College degree	4.13 ± 0.50	
		Bachelor’s degree and above	4.03 ± 0.51	
Sharing	Child Gender	Male	3.81 ± 0.59	−2.488 *
		Female	3.91 ± 0.59	
	Child age	3 years old	3.72 ± 0.58	10.787 ***
		4 years old	3.90 ± 0.58	
		5 years old	3.93 ± 0.61	
	Parents’ educational background	High school or vocational diploma	3.83 ± 0.60	1.286
		College degree	3.90 ± 0.60	
		Bachelor’s degree and above	3.84 ± 0.59	
Comforting	Child Gender	Male	3.79 ± 0.59	−1.685
		Female	3.86 ± 0.55	
	Child age	3 years old	3.69 ± 0.48	10.296 ***
		4 years old	3.89 ± 0.60	
		5 years old	3.86 ± 0.61	
	Parents’ educational background	High school or vocational diploma	3.78 ± 0.55	1.237
		College degree	3.86 ± 0.57	
		Bachelor’s degree and above	3.82 ± 0.58	
Helping	Child Gender	Male	3.92 ± 0.64	−1.319
		Female	3.97 ± 0.63	
	Child age	3 years old	3.88 ± 0.64	1.960
		4 years old	3.97 ± 0.61	
		5 years old	3.97 ± 0.65	
	Parents’ educational background	High school or vocational diploma	3.82 ± 0.66	6.462 **
		College degree	4.03 ± 0.63	
		Bachelor’s degree and above	3.96 ± 0.62	
Public Virtue	Child Gender	Male	4.07 ± 0.57	−2.462 *
		Female	4.17 ± 0.59	
	Child age	3 years old	4.03 ± 0.59	5.492 **
		4 years old	4.12 ± 0.60	
		5 years old	4.19 ± 0.54	
	Parents’ educational background	High school or vocational diploma	4.03 ± 0.62	4.573 *
		College degree	4.19 ± 0.58	
		Bachelor’s degree and above	4.12 ± 0.55	

Note. * *p* < 0.05, ** *p* < 0.01, *** *p* < 0.001.

**Table 4:** Descriptive Statistics and Correlation Analysis of Variables (*N* = 869).

Variable	*M ± SD*	1	2	3	4	5	6
1. Indulgent parenting style	1.82 ± 0.88	1	—	—	—	—	—
2. Democratic parenting style	4.11 ± 0.69	−0.269 **	1	—	—	—	—
3. Permissive parenting style	2.07 ± 0.93	0.558 **	−0.359 **	1	—	—	—
4. Authoritarian parenting style	2.49 ± 1.01	0.369 **	−0.151 **	0.428 **	1	—	—
5. Inconsistent parenting style	3.11 ± 1.07	0.426 **	−0.289 **	0.574 **	0.449 **	1	—
6. Children’s prosocial behavior	3.97 ± 0.81	−0.219 **	0.331 **	−0.267 **	−0.160 **	−0.231 **	1
7. Children’s emotional intelligence	4.63 ± 1.00	−0.165 **	0.332 **	−0.204 **	−0.145 **	−0.181 **	0.605 **

Note. ** *p* < 0.01.

**Table 5:** Intermediate Model Fit Indices.

Models	*χ*^2^/*df*	RMSEA	GFI	NFI	IFI	TLI	CFI	SRMR
Indulgent parenting style → CEI → CPB	4.606	0.064	0.939	0.917	0.934	0.921	0.934	0.027
Democratic parenting style → CEI → CPB	4.486	0.063	0.941	0.921	0.937	0.925	0.937	0.027
Permissive parenting style → CEI → CPB	4.444	0.063	0.942	0.920	0.937	0.925	0.937	0.027
Authoritarian parenting style → CEI → CPB	4.591	0.064	0.940	0.917	0.934	0.921	0.934	0.028
Inconsistent parenting style → CEI → CPB	4.434	0.063	0.942	0.920	0.937	0.925	0.937	0.027

**Table 6:** Mediating Effect of Children’s Emotional Intelligence on Parenting Styles and Children’s Prosocial Behavior.

Path	Parameter	Estimate	95% CI		*p*	Effect Size Proportion
			Lower Limit	Upper Limit		
Indulgent parenting style → CEI → CPB	Indirect effect	−0.118	−0.165	0.070	0.001	51.5%
	Direct effect	−0.111	−0.171	−0.059	0.001	48.5%
	Total effect	−0.229	−0.300	−0.154	0.001	
Democratic parenting style → CEI → CPB	Indirect effect	0.214	0.164	0.269	0.001	61.1%
	Direct effect	0.136	0.069	0.202	0.001	38.9%
	Total effect	0.350	0.269	0.425	0.001	
Permissive parenting style → CEI → CPB	Indirect effect	−0.142	−0.192	−0.095	0.001	49.0%
	Direct effect	−0.148	−0.204	−0.096	0.001	51.0%
	Total effect	−0.290	−0.357	−0.216	0.002	
Authoritarian parenting style → CEI → CPB	Indirect effect	−0.118	−0.165	−0.069	0.001	70.2%
	Direct effect	−0.050	−0.116	0.007	0.092	29.8%
	Total effect	−0.168	−0.238	−0.092	0.002	
Inconsistent parenting style → CEI → CPB	Indirect effect	−0.127	−0.175	−0.080	0.001	52.3%
	Direct effect	−0.116	−0.184	−0.059	0.001	47.7%
	Total effect	−0.243	−0.321	−0.161	0.001	

Note. Children’s emotional intelligence = CEI, Children’s prosocial behavior = CPB.

## Data Availability

The data that support the findings of this study are available on request from the corresponding author.

## Abbreviations

The following abbreviations are used in this manuscript:

SEM	Structural equation modeling
VIF	Variance inflation factor
